# Heart Failure with Preserved Ejection Fraction as a Multisystem Syndrome: A Mechanistically Anchored Endotype Framework for Precision Therapy

**DOI:** 10.3390/jcm15114159

**Published:** 2026-05-28

**Authors:** Beata Krasińska, Zbigniew Krasiński, Tomasz Urbanowicz

**Affiliations:** 1 Department of Hypertensiology, Angiology, and Internal Medicine, Poznan University of Medical Sciences, 61-701 Poznań, Poland; beata.bkrasinska@gmail.com; 2 Department of Vascular, Endovascular Surgery, Angiology and Phlebology, Poznan University of Medical Sciences, 61-701 Poznań, Poland; zbigniew.krasinski@gmail.com; 3 Cardiac Surgery and Transplantology Department, Poznan University of Medical Sciences, 61-701 Poznań, Poland

**Keywords:** HFpEF, heart failure with preserved ejection fraction, endotypes, precision medicine, cardiorenal syndrome, metabolic inflammation, myocardial fibrosis, SGLT2 inhibitors, GLP-1 receptor agonists, biomarker-guided therapy

## Abstract

Heart failure with preserved ejection fraction (HFpEF) accounts for approximately half of all heart failure cases worldwide and remains associated with substantial morbidity, mortality, and limited therapeutic efficacy. This persistent therapeutic gap reflects not only clinical heterogeneity but also a fundamental conceptual limitation: HFpEF has been approached as a myocardial disorder despite mounting evidence that it represents a multisystem syndrome. In this perspective, we integrate current pathophysiological and clinical evidence into a mechanistically anchored endotype framework based on dominant biological axes. We propose four overlapping endotypes—cardiorenal, fibrotic–inflammatory, metabolic–adiposity, and microvascular–energetic—and align contemporary and emerging therapies with these domains. We further outline a pragmatic roadmap for biomarker-guided stratification and propose principles for next-generation clinical trial design based on mechanistic enrichment. This framework provides a clinically actionable structure for biomarker-guided stratification and supports mechanism-based therapeutic selection in HFpEF. Aligning dominant biological drivers with targeted interventions generates testable hypotheses for endotype-enriched clinical trials and precision treatment strategies. Prospective validation of this approach may facilitate a transition from uniform management toward individualized, biology-driven care.

## 1. Introduction

Heart failure with preserved ejection fraction (HFpEF) now represents approximately 50% of all heart failure cases and continues to increase in prevalence due to population aging and the growing burden of cardiometabolic disease [1,2,3]. Patients are typically older and multimorbid, with high rates of hospitalization and mortality comparable to those observed in heart failure with reduced ejection fraction (HFrEF) [4,5].

Despite its prevalence, therapeutic progress in HFpEF has been modest. In extensive investigation, most large-scale clinical trials in HFpEF have demonstrated neutral or only modest effects on primary outcomes, with relative risk reductions typically in the range of 10–20% and substantial heterogeneity across subgroups. This variability underscores the likelihood that treatment effects are diluted within biologically heterogeneous populations, in which the targeted pathway may not represent the dominant disease driver in a significant proportion of patients.

While sodium–glucose cotransporter 2 (SGLT2) inhibitors have demonstrated consistent benefit [6,7], most pharmacological strategies targeting isolated pathways have yielded neutral or modest effects [8,9].

A major limitation may lie in the prevailing conceptual framework [10]. HFpEF has traditionally been defined by diastolic dysfunction; however, this paradigm fails to capture the systemic nature of the disease. Increasing evidence supports a model in which HFpEF arises from interacting biological domains, including metabolic inflammation, endothelial dysfunction, renal impairment, and altered volume homeostasis [11,12,13,14].

While prior conceptual models have emphasized individual pathways or phenotypic clustering, they have not consistently translated into clinically actionable strategies. The present framework differs in that it explicitly links dominant biological mechanisms to measurable biomarkers and corresponding therapeutic domains, thereby providing a structured basis for mechanism-guided treatment selection rather than relying solely on descriptive classification.

Unlike machine-learning phenogroups, which are cohort-dependent and often non-reproducible, our framework is mechanistically anchored and clinically portable. The previous efforts to stratify HFpEF have largely relied on data-driven phenogrouping or comorbidity-based classifications; these approaches are inherently cohort-dependent and often lack direct therapeutic implications. In contrast, the present framework is mechanism-first and explicitly links dominant biological processes to measurable biomarkers and therapeutic domains. By prioritizing pathophysiological drivers rather than statistical clustering, this model is designed to be portable across clinical settings and to support treatment selection at the individual patient level. This distinction is essential, as clinically meaningful stratification in HFpEF must ultimately inform—not merely describe—therapeutic decision-making.

Despite decades of investigation, HFpEF remains one of the few major cardiovascular syndromes without a broadly effective disease-modifying therapy beyond SGLT2 inhibition. Event rates remain high, with hospitalization and mortality comparable to those observed in HFrEF, underscoring a persistent gap between pathophysiological insight and therapeutic translation. This disconnect suggests that the central challenge in HFpEF is not the absence of therapeutic targets, but rather the failure to align interventions with dominant biological drivers within heterogeneous patient populations.

This manuscript should be interpreted as a mechanistically oriented perspective rather than a systematic review. The objective was not to perform formal evidence synthesis using PRISMA methodology or quantitative evidence grading, but rather to integrate contemporary pathophysiological, translational, and clinical evidence into a clinically actionable conceptual framework. References were selected on the basis of mechanistic relevance, landmark clinical data, and applicability to precision-oriented HFpEF management.

## 2. From Ventricular Dysfunction to Systemic Disease

The systemic paradigm of HFpEF was articulated by Paulus and Tschöpe, who proposed that comorbidity-driven inflammation leads to coronary microvascular dysfunction and myocardial stiffening [15,16]. Subsequent studies using phenogrouping and machine-learning approaches have confirmed the presence of biologically distinct HFpEF subgroups characterized by distinct clinical trajectories and therapeutic responses [17,18,19,20].

Within this framework, myocardial dysfunction is often a downstream manifestation of systemic dysregulation rather than the primary driver. This perspective helps explain the limited efficacy of therapies targeting isolated cardiac pathways and the relative success of treatments with broader systemic effects. Notably, phenogrouping analyses have consistently identified subpopulations characterized by metabolic, cardiorenal, and inflammatory signatures, each associated with distinct clinical trajectories and differential responses to therapy, reinforcing the concept that HFpEF is not a single entity but a spectrum of biologically distinct conditions.

Sex and aging likely play central roles in determining HFpEF biology and endotype expression [21,22]. HFpEF disproportionately affects older women, in whom age-related vascular stiffening, altered ventricular–vascular coupling, adipose redistribution, and endothelial dysfunction may interact with hormonal transitions following menopause. In contrast, male HFpEF phenotypes may exhibit greater ischemic burden and myocardial remodeling. Aging itself promotes chronic low-grade inflammation, mitochondrial dysfunction, microvascular rarefaction, and impaired reserve capacity across multiple organ systems, thereby amplifying susceptibility to multisystem dysregulation. These observations further support the concept that HFpEF represents a systemic syndrome whose biological expression is shaped by both sex-specific and age-dependent mechanisms.

Unlike prior frameworks based on phenogrouping or comorbidity clustering, which are inherently dependent on specific datasets and may lack reproducibility across populations, the present model is explicitly mechanism-driven and designed for clinical portability. By linking dominant biological processes to measurable biomarkers and therapeutic domains, this framework aims not only to describe heterogeneity but to operationalize it in a manner that directly informs treatment selection.

## 3. A Mechanistically Anchored Endotype Framework

Precise prevalence varies across cohorts; emerging data suggest that the metabolic–adiposity endotype may account for the majority of contemporary HFpEF cases, particularly in Western populations characterized by high rates of obesity and diabetes. Cardiorenal phenotypes are also highly prevalent and associated with adverse outcomes, while fibrotic–inflammatory and microvascular–energetic patterns may represent more specific, but clinically significant subgroups. Importantly, these endotypes frequently coexist, reinforcing the need for multidimensional rather than categorical classification.

Rather than viewing HFpEF as a single disease with variable presentation, we propose a framework based on dominant biological drivers—endotypes that reflect underlying pathophysiology rather than surface phenotype. These domains—cardiorenal, fibrotic–inflammatory, metabolic–adiposity, and microvascular–energetic—are not discrete categories but interacting axes along which individual patients can be positioned. This approach does not replace clinical phenotyping but refines it, providing a mechanistic structure that may better inform therapeutic decision-making.

We propose four principal HFpEF endotypes defined by dominant biological drivers. These endotypes are overlapping and dynamic rather than mutually exclusive. The integration of biological mechanisms, clinical features, biomarkers, and therapeutic strategies across endotypes is summarized in Table 1.

Most patients exhibit hybrid biological signatures rather than isolated endotypes, reinforcing the need for multidimensional rather than binary classification.

**Figure 1:**
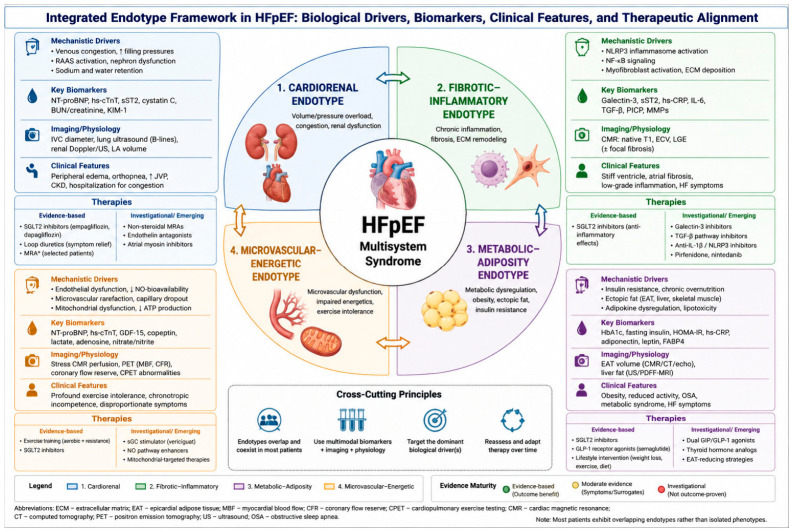
Integrated endotype frameworks in HFpEF.

The integrated relationship between the four proposed HFpEF endotypes, their dominant biological drivers, representative biomarkers, clinical phenotypes, and therapeutic alignment is summarized in Figure 1. This schematic emphasizes that the endotypes are not mutually exclusive categories but overlapping biological axes that may coexist within individual patients. The figure also distinguishes therapies supported by clinical evidence from investigational or emerging strategies, highlighting the need to match treatment selection to the dominant biological driver rather than applying uniform therapy across all HFpEF patients.

These endotypes should not be interpreted as isolated entities but as components of an interconnected biological network. For example, metabolic inflammation may exacerbate endothelial dysfunction, which in turn promotes microvascular rarefaction and myocardial fibrosis. Similarly, renal sodium retention can amplify systemic inflammation and hemodynamic stress, creating self-reinforcing pathological loops. This network perspective supports the rationale for combinatorial therapeutic strategies targeting multiple axes simultaneously.

The proposed endotype framework demonstrates convergence with previously described phenogrouping strategies while offering greater mechanistic interpretability. For example, clusters characterized by obesity, diabetes, and systemic inflammation correspond closely to the metabolic–adiposity endotype, whereas phenogroups defined by renal dysfunction and volume overload align with the cardiorenal axis. By mapping data-driven phenotypes onto biologically defined mechanisms, this framework seeks to bridge descriptive classification and therapeutic applicability, thereby enhancing clinical translatability.

## 4. Endotypes

1. Cardiorenal Sodium–Volume Dysregulation

Mechanism: impaired renal sodium handling and venous congestion.

Evidence: congestion-driven HFpEF phenotypes are associated with worse outcomes and a strong response to natriuretic therapies [23,24,25].

Therapeutic alignment: SGLT2 inhibitors, diuretics, renin–angiotensin–aldosterone axis (RAAS) modulation.

Clinically, this endotype is often reflected in patients presenting with recurrent congestion, renal dysfunction, and a pronounced response to natriuretic therapies.

2. Fibrotic–Inflammatory Endotype

Mechanism: Chronic inflammation and extracellular matrix expansion.

Evidence: Elevated galectin-3 (Gal-3) and soluble interleukin-1 receptor-like 1 (sST2) are associated with adverse outcomes and myocardial fibrosis [26,27,28]. At the molecular level, the fibrotic–inflammatory endotype is increasingly linked to persistent activation of innate immune signaling pathways, particularly NLRP3 inflammasome activation and downstream NF-κB-mediated transcriptional responses [29]. Chronic metabolic stress, oxidative injury, venous congestion, and endothelial dysfunction may collectively sustain low-grade sterile inflammation, promoting macrophage activation, fibroblast proliferation, extracellular matrix expansion, and progressive myocardial stiffening. This inflammatory–fibrotic coupling may represent a critical mechanistic bridge between systemic comorbidity burden and structural cardiac remodeling in HFpEF. Importantly, the persistence of unresolved inflammatory activation despite standard neurohormonal therapy may help explain the limited efficacy of conventional heart failure strategies in this subgroup and reinforce the rationale for targeted anti-inflammatory and antifibrotic approaches.

Therapeutic alignment: Mineralocorticoid receptor antagonists (including finerenone) and emerging antifibrotic therapies. This pattern is frequently observed in patients with systemic inflammatory burden and progressive structural remodeling, even in the absence of overt volume overload.

3. Metabolic–Adiposity Endotype

Mechanism: Adipose-driven inflammation and metabolic dysfunction.

Evidence: Obesity-related HFpEF is characterized by epicardial fat expansion, insulin resistance, and systemic inflammation [30,31,32].

Therapeutic alignment: GLP-1 receptor agonists and dual incretin therapies (e.g., semaglutide; STEP-HFpEF trial) [33,34,35].

In clinical practice, this endotype corresponds to the obese, insulin-resistant HFpEF phenotype characterized by reduced exercise capacity and systemic metabolic dysregulation.

In this context, the limited success of prior interventions may be understood not as a failure of individual therapies but as a mismatch between therapeutic targets and the dominant biological drivers present in heterogeneous patient populations.

4. Microvascular–Energetic Endotype

Mechanism: Endothelial dysfunction and impaired myocardial energetics.

Evidence: Reduced nitric oxide bioavailability and mitochondrial dysfunction contribute to exercise intolerance in HFpEF [36,37]. Emerging evidence suggests that impaired myocardial energetics and peripheral oxygen utilization may represent central determinants of exercise intolerance in HFpEF [38,39]. Beyond nitric oxide pathway dysregulation, mitochondrial dysfunction, reduced oxidative phosphorylation efficiency, skeletal muscle abnormalities, and impaired microvascular reserve likely contribute to reduced functional capacity. Therapeutically, this has renewed interest in interventions extending beyond vasodilation alone, including exercise training protocols, metabolic modulation, and agents influencing soluble guanylate cyclase signaling such as vericiguat. In parallel, experimental data suggest that SGLT2 inhibitors may exert favorable mitochondrial and energetic effects independent of their renal actions, further supporting the interconnected biology underlying this endotype [40,41].

Cardiopulmonary exercise testing (CPET) represents a particularly valuable tool within this endotype, as exercise intolerance is often disproportionate to resting hemodynamic abnormalities [42]. Parameters such as peak VO2, ventilatory efficiency (VE/VCO2 slope), chronotropic response, and exercise oscillatory ventilation may provide objective insight into impaired peripheral oxygen utilization, abnormal energetic reserve, and microvascular dysfunction. Beyond its diagnostic value, CPET may also support longitudinal assessment of therapeutic response and refinement of mechanism-guided stratification strategies in HFpEF.

Therapeutic alignment: NO pathway modulation, and mitochondrial-targeted therapies (investigational).

**Figure 2:**
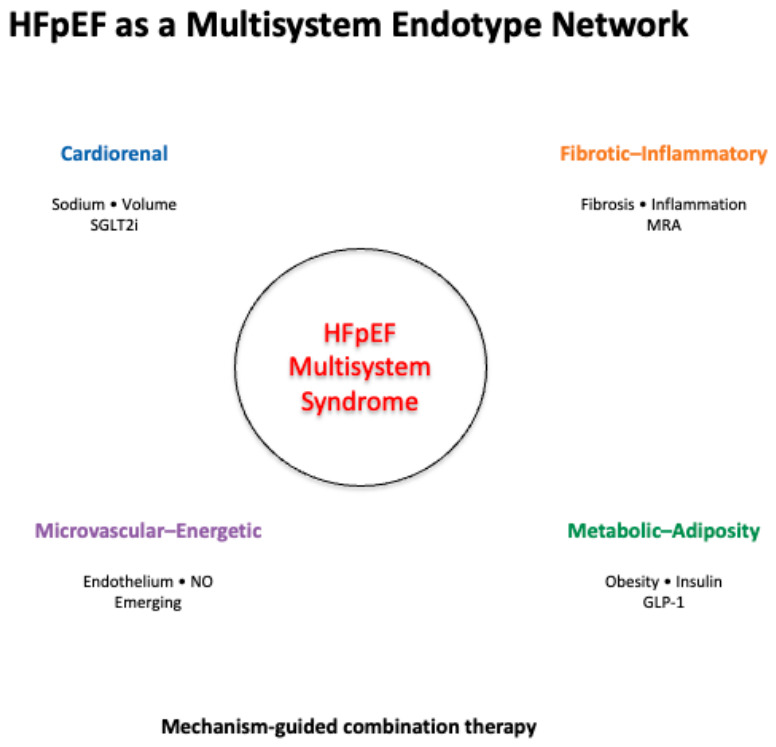
Systems biology framework of HFpEF: Endotypes and therapeutic targets.

Figure 2 presents a schematic systems biology framework for HFpEF and its therapeutic targets.

Clinical implementation note: Endotype assignment should be guided by dominant clinical features supported by routinely available biomarkers. At present, no universally validated threshold system exists for HFpEF endotype classification; however, pragmatic indicators may assist initial stratification. Examples include elevated natriuretic peptides consistent with hemodynamic congestion, impaired renal function (eGFR < 60 mL/min/1.73 m^2^), elevated HbA1c or insulin resistance indices in metabolic phenotypes, and increased galectin-3 or sST2 concentrations in fibrotic–inflammatory disease. CPET abnormalities and imaging evidence of epicardial adiposity or myocardial fibrosis may further refine classification. Importantly, these parameters should currently be interpreted as biologically informative rather than diagnostically definitive, pending prospective validation.

HFpEF is conceptualized as a multisystem syndrome arising from four interacting biological domains: cardiorenal sodium–volume dysregulation, fibrotic–inflammatory remodeling, metabolic–adiposity-driven dysfunction, and microvascular–energetic impairment. These domains converge to produce the clinical HFpEF phenotype through shared downstream effects, including elevated filling pressures and reduced functional capacity. Therapeutic strategies align with dominant biological axes, supporting a mechanism-guided and potentially combinatorial treatment approach.

## 5. Reinterpreting Therapeutic Success Through Biology

Several large HFpEF trials targeting isolated neurohormonal or hemodynamic pathways—including PARAGON-HF and TOPCAT [8,43]—have yielded neutral or modest results despite a strong mechanistic rationale. These findings are often attributed to heterogeneity in study populations or to limitations in trial design; however, they may more fundamentally reflect a mismatch between the biological targets of these interventions and the dominant disease drivers present within enrolled cohorts. In a syndrome characterized by overlapping and dynamic pathophysiological processes, therapies directed at a single axis are unlikely to demonstrate uniform benefit across unselected populations. This perspective underscores the need for mechanistic enrichment strategies in future trial design.

Importantly, several therapies that have failed to demonstrate benefit in HFpEF—including nitric oxide–cGMP pathway modulators and neprilysin inhibition in unselected populations—may not be inherently ineffective, but rather insufficiently matched to the dominant biological processes present within trial cohorts. This interpretation shifts the focus from drug efficacy in isolation to the alignment between therapeutic mechanism and disease biology.

The repeated neutral or modest outcomes of HFpEF trials are often attributed to heterogeneity or suboptimal design. While both factors are relevant, they obscure a more fundamental issue: most interventions have targeted isolated pathways within a disease that is inherently multisystem. Therapies directed at ventricular mechanics or single neurohormonal axes are unlikely to succeed when the dominant drivers lie outside the myocardium [44]. In this light, the limited efficacy of many agents reflects not pharmacological failure, but biological misalignment.

Viewed through an endotype-based lens, the neutral or modest outcomes of major HFpEF trials may reflect inadequate biological alignment rather than a lack of therapeutic efficacy. In unselected trial populations, such alignment is unlikely to occur consistently, resulting in attenuation of observable treatment effects at the cohort level.

SGLT2 Inhibitors

Large, randomized trials, including EMPEROR-Preserved (Empagliflozin Outcome Trial in Patients With Chronic Heart Failure With Preserved Ejection Fraction) and DELIVER (Dapagliflozin Evaluation to Improve the LIVEs of Patients With Preserved Ejection Fraction Heart Failure), have demonstrated consistent reductions in heart failure hospitalization across the HFpEF spectrum [5,6]. These agents appear to act at the level of systemic regulation—modulating renal sodium handling, improving energetic efficiency, and attenuating inflammation. The broad efficacy of SGLT2 inhibitors across HFpEF populations may derive from their simultaneous modulation of several interconnected biological axes rather than from selective targeting of a single pathway. Beyond natriuresis and reduction in filling pressures, these agents influence myocardial energetics, systemic inflammation, endothelial function, renal hemodynamics, and metabolic efficiency [45,46]. This multidomain activity makes SGLT2 inhibition uniquely aligned with the network-based biology of HFpEF and may explain why these therapies have demonstrated greater consistency than interventions directed at isolated molecular pathways.

Their efficacy may therefore reflect alignment with the multidimensional biology of HFpEF, rather than superiority within any single pathway. This distinction is critical, as it suggests that future therapies should be evaluated not by their molecular target, but by their integration within disease networks. SGLT2 inhibitors not only improve renal sodium handling, but also reduce congestion, enhance myocardial energetics, and attenuate inflammation [47,48]. Their pleiotropic effects likely explain their broad efficacy. Importantly, the therapeutic domains outlined in this framework differ substantially in evidentiary maturity. SGLT2 inhibitors currently represent the only therapeutic class with consistent randomized evidence demonstrating reduction in HF hospitalization across the HFpEF spectrum. Incretin-based therapies have demonstrated robust improvements in symptoms, exercise capacity, and quality of life in obesity-related HFpEF, although long-term outcome data remain limited. In contrast, antifibrotic and microvascular-targeted strategies remain investigational and are currently supported primarily by mechanistic studies, biomarker analyses, and early-phase clinical trials. Distinguishing between validated and emerging therapeutic domains is essential to avoid overinterpretation of the framework and to appropriately position mechanistically promising but unproven interventions.

Mineralocorticoid Receptor Antagonism

Finerenone and related agents target inflammation and fibrosis at the tissue level. While clinical benefits are modest, they reinforce the importance of extracellular matrix remodeling in HFpEF [49,50,51].

Incretin-Based Therapies

The STEP-HFpEF trial demonstrated that semaglutide improves symptoms and functional capacity in obese HFpEF patients [15]. These therapies act upstream by modifying adiposity and systemic inflammation, supporting a shift toward targeting disease drivers rather than cardiac consequences.

**Figure 3:**
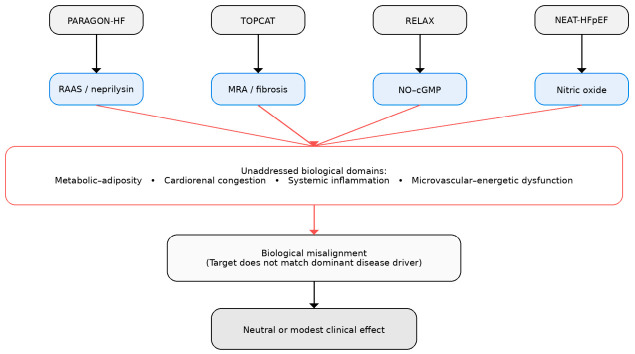
Biological misalignment as a determinant of neutral outcomes in HFpEF clinical trials. Several major HFpEF trials have targeted individual pathophysiological pathways, including neurohormonal activation and nitric oxide signaling, yet have yielded neutral or modest clinical effects. This schematic illustrates how such outcomes may reflect a mismatch between the targeted mechanism and the dominant biological drivers present within heterogeneous patient populations. In contrast, therapies with broader, multisystem effects may achieve greater efficacy by aligning with multiple interacting disease pathways.

A unifying explanation for the neutral or modest outcomes observed in multiple HFpEF trials is the concept of biological misalignment. In many studies, therapeutic interventions have targeted single pathophysiological pathways—such as neurohormonal activation or nitric oxide signaling—within populations characterized by substantial biological heterogeneity. Such approaches may fail not because the targeted mechanisms are irrelevant, but because they do not represent the dominant drivers of disease in a significant proportion of enrolled patients. For example, modulation of the NO–cGMP pathway may have limited impact in individuals whose disease is primarily driven by metabolic or cardiorenal dysfunction, while antifibrotic strategies may be insufficient in the absence of concurrent congestion or systemic inflammation. In contrast, therapies with pleiotropic, multisystem effects—such as SGLT2 inhibitors—may achieve broader efficacy by simultaneously engaging multiple biological axes. This perspective reframes prior “negative” trials not as failures of pharmacology, but as evidence of the need for mechanism-aligned therapeutic strategies and biologically enriched trial design, shown in Figure 3.

## 6. Toward Rational Combination Therapy

From a systems perspective, combination therapy in HFpEF should be conceptualized not as additive pharmacology but as coordinated modulation of interacting biological networks. For example, simultaneous targeting of cardiorenal congestion and metabolic inflammation may interrupt reinforcing feedback loops that sustain disease progression. Such approaches mirror strategies successfully employed in oncology and infectious disease, where multidimensional interventions are required to address complex pathophysiology.

A mechanistically informed approach also enables the definition of prototype combination strategies targeting complementary biological axes. For example, in patients with coexisting cardiorenal and metabolic features, the combination of an SGLT2 inhibitor with an incretin-based therapy may simultaneously address volume dysregulation, insulin resistance, and systemic inflammation. Similarly, the addition of mineralocorticoid receptor antagonism in patients with fibrotic–inflammatory features may further modulate extracellular matrix remodeling. Such multidimensional strategies aim not merely to add pharmacological effects but to disrupt reinforcing pathological networks.

The modest effect sizes observed in HFpEF trials likely reflect the limitations of single-pathway interventions in a multisystem disease. A mechanistically guided approach suggests combining therapies targeting complementary axes:

- Cardiorenal: SGLT2 inhibitors;
- Fibrotic–inflammatory: Mineralocorticoid receptor antagonists;
- Metabolic: Incretin-based therapies.

Such strategies should be individualized based on dominant endotype features rather than applied uniformly.

Operationalizing Endotype-Based Care

Endotype classification should be viewed as dynamic rather than static. The dominant biological drivers in an individual patient may evolve over time in response to disease progression, comorbidity burden, and therapeutic intervention. This temporal variability necessitates periodic reassessment and supports a longitudinal, adaptive approach to HFpEF management.

For this framework to be clinically actionable, endotype assignment must be reproducible and pragmatic. Rather than relying on complex or experimental assays, an initial implementation can be based on the integration of routinely available clinical features, standard laboratory biomarkers, and accessible imaging data. Importantly, endotypes should be interpreted as dominant biological tendencies rather than mutually exclusive categories, allowing for the identification of primary and secondary drivers within individual patients. This layered approach reflects the multidimensional nature of HFpEF while maintaining clinical feasibility.

Clinical implementation requires reproducible stratification. A pragmatic approach may include:

1. Multimodal profiling (clinical, biochemical, imaging);
2. Endotype assignment based on dominant biological signals;
3. Iterative therapeutic adjustment.

At present, this approach remains investigational and requires validation in prospective studies.

## 7. Clinical Algorithm for Endotype-Guided Management in HFpEF

**Figure 4:**
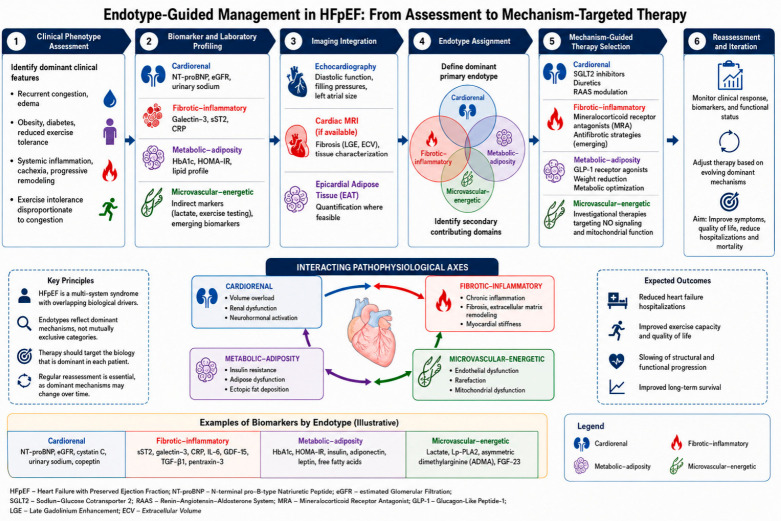
Endotype-guided management in HFpEF.

We propose a clinical algorithm for endotype-guided HFpEF management as described step-by-step below and shown in Figure 4.

Step 1: Clinical Phenotype Assessment
Identify dominant clinical features:

− Recurrent congestion, edema → suspect cardiorenal axis;
− Obesity, diabetes, reduced exercise tolerance → metabolic axis;
− Systemic inflammation, cachexia, progressive remodeling → fibrotic–inflammatory axis;
− Exercise intolerance disproportionate to congestion → microvascular–energetic axis.

Step 2: Biomarker and Laboratory Profiling

− Cardiorenal: NT-proBNP, eGFR, urinary sodium;
− Fibrotic–inflammatory: Galectin-3, sST2, CRP;
− Metabolic: HbA1c, HOMA-IR, lipid profile;
− Microvascular–energetic: Indirect markers (lactate, exercise testing), emerging biomarkers.

Step 3: Imaging Integration

− Echocardiography: Diastolic function, filling pressures;
− Cardiac MRI (if available): Fibrosis, tissue characterization;
− Assessment of epicardial adipose tissue (EAT) where feasible.

Epicardial adipose tissue may be quantified using multiple imaging modalities, including echocardiography, cardiac magnetic resonance imaging, and computed tomography. Echocardiography offers pragmatic bedside assessment, whereas CMR and CT provide superior volumetric characterization and tissue resolution [52,53]. Integration of epicardial adipose tissue (EAT) imaging into multimodal HFpEF assessment may improve identification of metabolically driven phenotypes and facilitate longitudinal evaluation of adiposity-targeted therapies.

Step 4: Endotype Assignment

− Define dominant (primary) endotype;
− Identify secondary contributing domains.

Step 5: Mechanism-Guided Therapy Selection

− Cardiorenal: SGLT2 inhibitors, diuretics, RAAS modulation;
− Fibrotic–inflammatory: Mineralocorticoid receptor antagonists, antifibrotic strategies (emerging);
− Metabolic–adiposity: GLP-1 receptor agonists, weight reduction strategies;
− Microvascular–energetic: Investigational therapies targeting NO signaling and mitochondrial function.

Step 6: Reassessment and Iteration

− Monitor clinical response and biomarkers;
− Adjust therapy based on evolving dominant mechanisms.

Biomarker Thresholds for Initial Endotype Assignment

To improve the clinical applicability of the proposed framework, pragmatic biomarker ranges may be used to support provisional endotype assignment, although these thresholds should currently be interpreted as biologically informative rather than formally validated diagnostic criteria. The purpose of these cutoffs is not to establish rigid categorical definitions, but to facilitate reproducible mechanism-oriented stratification in clinical practice and future biomarker-enriched trials.

For the cardiorenal endotype, features supporting dominant sodium–volume dysregulation may include NT-proBNP concentrations > 300 pg/mL in sinus rhythm or >600–900 pg/mL in atrial fibrillation, estimated glomerular filtration rate (eGFR) < 60 mL/min/1.73 m^2^, urinary sodium < 70 mmol/L following diuretic administration, and persistent clinical congestion.

The fibrotic–inflammatory endotype may be suggested by galectin-3 concentrations > 17.8 ng/mL, sST2 > 35 ng/mL, elevated high-sensitivity C-reactive protein (>3 mg/L), or imaging evidence of diffuse myocardial fibrosis on cardiac magnetic resonance imaging.

The metabolic–adiposity endotype may be identified in patients with body mass index ≥ 30 kg/m^2^, HbA1c ≥ 6.5%, HOMA-IR > 2.5–3.0, increased epicardial adipose tissue thickness (>5 mm by echocardiography), or established insulin resistance/metabolic syndrome.

The microvascular–energetic endotype may be suspected in patients with disproportionate exercise intolerance despite modest resting congestion, peak VO2 < 14–16 mL/kg/min, VE/VCO2 slope > 34 on cardiopulmonary exercise testing, elevated exercise lactate levels, or evidence of impaired peripheral oxygen extraction.

Importantly, overlap between domains is expected, and patients may fulfill criteria for multiple endotypes simultaneously. In such cases, classification should prioritize the dominant biological driver most closely associated with symptoms, congestion burden, or disease progression.

Table 2 presents the biomarker thresholds supporting the assignment of HFpEF endotypes.

From a practical standpoint, implementation of this algorithm does not require advanced or experimental diagnostics. An initial endotype assignment can be achieved using standard clinical assessment, routine laboratory testing, and echocardiographic evaluation. Advanced imaging or biomarker panels may refine classification where available, but are not essential for initial stratification. This pragmatic approach facilitates integration into routine clinical workflows while preserving the conceptual integrity of mechanism-based care.

## 8. Implications for Clinical Trial Design

Traditional HFpEF trials are limited by biological heterogeneity and endpoint misalignment. Future trials should incorporate:

- Mechanistic enrichment using biomarkers;
- Adaptive designs;
- Biology-aligned endpoints (e.g., functional capacity, recurrent events).

Such strategies are standard in oncology and may be necessary to unlock therapeutic advances in HFpEF.

This paradigm mirrors the evolution of precision oncology, where therapeutic success increasingly depends on biomarker-defined patient selection rather than disease site alone. Strategies such as basket and umbrella trial designs, which allocate therapies based on molecular characteristics rather than traditional diagnostic categories, may provide a useful template for HFpEF. Applying similar principles could enable more efficient identification of treatment-responsive subgroups and accelerate therapeutic development in this biologically heterogeneous syndrome.

As an illustrative example, a biomarker-enriched trial targeting the metabolic–adiposity endotype could enroll patients with HFpEF and obesity (body mass index ≥ 30 kg/m^2^) with evidence of insulin resistance or type 2 diabetes. Similarly, a biomarker-enriched trial focused on the fibrotic–inflammatory endotype could selectively enroll patients with elevated galectin-3 or sST2 concentrations together with imaging evidence of myocardial fibrosis. Such a design would allow for targeted evaluation of antifibrotic or anti-inflammatory therapies using endpoints aligned with extracellular matrix remodeling, functional status, and recurrent HF hospitalization. In parallel, cardiorenal-enriched strategies could prioritize patients with persistent congestion, impaired renal function, and elevated natriuretic peptides to evaluate multidimensional decongestive approaches.

Randomization to incretin-based therapy versus standard care could be evaluated using endpoints aligned with the dominant biology of this subgroup, such as functional capacity, symptom burden, and quality of life, in addition to conventional clinical outcomes. Such designs may increase the likelihood of detecting clinically meaningful treatment effects within biologically coherent populations.

## 9. Clinical Perspective

From a clinical perspective, the value of this framework lies less in its taxonomy and more in its ability to reorient therapeutic thinking. HFpEF management often defaults to a uniform approach despite marked biological heterogeneity between patients. In practice, clinicians already recognize patterns—patients driven by congestion, others by obesity and metabolic dysfunction, or those with prominent inflammatory or vascular features—but lack a structured way to translate these observations into targeted therapy. An endotype-based framework provides that missing link. It does not require immediate adoption of complex biomarker panels but rather encourages a more deliberate alignment between dominant clinical features and therapeutic choices. In this sense, it formalizes what experienced clinicians already do intuitively, while offering a pathway toward more precise and reproducible care.

For example, a patient with obesity, insulin resistance, and reduced functional capacity may derive greater benefit from incretin-based therapy, whereas a patient with recurrent congestion and renal impairment may be more appropriately managed with therapies targeting cardiorenal mechanisms.

In routine practice, this framework supports a shift from uniform treatment strategies toward mechanism-oriented care. Clinicians may use readily available clinical features and standard biomarkers to identify dominant biological drivers and select therapies accordingly. This approach encourages early consideration of combination therapy targeting complementary pathways and emphasizes the need for periodic reassessment as disease biology evolves.

### Limitations

Several important limitations should be acknowledged. First, the proposed framework is conceptual and has not yet been prospectively validated in clinical trials. Second, substantial overlap between endotypes may limit the precision of classification in individual patients. Third, many candidate biomarkers lack standardized thresholds and may be influenced by comorbid conditions, reducing specificity. Fourth, the framework simplifies a highly complex biological system and may not fully capture emerging mechanisms, such as right ventricular dysfunction, lymphatic congestion, or alterations in the endothelial glycocalyx. Finally, implementation in routine practice may be constrained by resource availability and variability in access to advanced diagnostics.

Furthermore, the proposed framework may oversimplify a highly complex and continuously interacting biological system, and misclassification remains possible, particularly in the absence of standardized biomarker thresholds or in patients with multiple co-dominant mechanisms. This highlights the need for iterative refinement and prospective validation.

## 10. Conclusions

HFpEF should no longer be approached as a uniform cardiac disorder defined by preserved ejection fraction, but as a syndrome arising from interacting biological systems. The implications are profound: effective treatment will not come from a single transformative agent, but from aligning therapy with the dominant mechanisms in each patient. A mechanistically anchored framework offers a path toward this goal—enabling more precise stratification, rational combination therapy, and more informative clinical trials. The challenge ahead is not simply to develop new drugs, but to redefine the disease they are intended to treat. Ultimately, progress in HFpEF will depend less on the discovery of a single transformative therapy and more on the ability to match existing and emerging treatments to the right biological context.

Three key implications emerge from this framework. First, HFpEF should be understood as a multisystem disorder rather than a primary myocardial disease. Second, the limited success of prior therapies may reflect biological misalignment rather than pharmacological inadequacy. Third, meaningful therapeutic progress will likely depend on aligning treatment strategies with dominant pathophysiological mechanisms at the individual patient level. Advancing this paradigm will require integrating mechanistic insights into both clinical practice and trial design.

The future of HFpEF management will likely depend not on identifying a universal therapy but on integrating mechanistic stratification, biomarker-guided treatment selection, and adaptive multidomain intervention strategies capable of addressing the biological complexity of the syndrome.

## Figures and Tables

**Table 1:** Endotype–therapy alignment matrix in HFpEF. This matrix integrates the four principal HFpEF endotypes with their corresponding biological mechanisms, clinical features, biomarkers, and therapeutic strategies. Evidence strength varies across domains, with robust randomized trial data supporting cardiorenal and metabolic interventions, moderate evidence for antifibrotic strategies, and emerging data for microvascular–energetic targeting. The table emphasizes that effective HFpEF management requires alignment between dominant biological drivers and therapeutic mechanisms rather than uniform treatment application.

Endotype	Core Mechanism	Dominant Clinical Phenotype	Representative Biomarkers	Therapeutic Alignment	Key Evidence Level
Cardiorenal sodium–volume	Renal sodium retention, venous congestion, altered volume distribution	Recurrent congestion, edema, renal dysfunction, elevated filling pressures	NT-proBNP/BNP, eGFR, urinary sodium, congestion indices	SGLT2 inhibitors, loop diuretics, RAAS modulation	Strong (RCT + observational)
Fibrotic–inflammatory	Chronic inflammation, extracellular matrix expansion, myocardial fibrosis	Progressive remodeling, systemic inflammation, cachexia (in advanced cases)	Galectin-3, sST2, CRP, PICP, PIIINP	MR antagonists (e.g., finerenone), antifibrotic therapies (emerging)	Moderate (observational + mechanistic)
Metabolic–adiposity	Insulin resistance, adipose inflammation, epicardial fat expansion	Obesity, diabetes, reduced exercise capacity, systemic metabolic dysfunction	HbA1c, HOMA-IR, leptin/adiponectin, EAT imaging	GLP-1 receptor agonists, dual incretins (e.g., semaglutide)	Strong (RCT for symptoms/function)
Microvascular–energetic	Endothelial dysfunction, impaired nitric oxide signaling, mitochondrial dysfunction	Exercise intolerance disproportionate to congestion, preserved resting hemodynamics	Indirect markers (lactate, exercise testing), emerging biomarkers	NO pathway modulation, mitochondrial-targeted therapies (investigational)	Limited (mechanistic + early-phase)

Abbreviations: BNP, B-type natriuretic peptide; CPET, cardiopulmonary exercise testing; CRP, C-reactive protein; EAT, epicardial adipose tissue; eGFR, estimated glomerular filtration rate; HbA1c, glycated hemoglobin; HOMA-IR, homeostatic model assessment of insulin resistance; NT-proBNP, N-terminal pro-B-type natriuretic peptide; PICP, procollagen type I C-terminal propeptide; PIIINP, procollagen type III N-terminal propeptide; RAAS, renin–angiotensin–aldosterone system; sST2, soluble suppression of tumorigenicity-2; SGLT2, sodium–glucose cotransporter 2.

**Table 2:** Biomarker thresholds supporting HFpEF endotype assignment.

Endotype	Suggested Operational Markers	Proposed Pragmatic Thresholds *	Dominant Therapeutic Direction
Cardiorenal	NT-proBNP, eGFR, urinary sodium	NT-proBNP > 300 pg/mL (SR), >600–900 pg/mL (AF); eGFR < 60 mL/min/1.73 m^2^; urinary sodium < 70 mmol/L	SGLT2 inhibitors, diuretics, RAAS modulation
Fibrotic–inflammatory	Galectin-3, sST2, hs-CRP	Galectin-3 > 17.8 ng/mL; sST2 > 35 ng/mL; hs-CRP > 3 mg/L	MRA/finerenone, antifibrotic strategies
Metabolic–adiposity	BMI, HbA1c, HOMA-IR, EAT	BMI ≥ 30 kg/m^2^; HbA1c ≥ 6.5%; HOMA-IR > 2.5–3.0; EAT > 5 mm	GLP-1 receptor agonists, weight reduction
Microvascular–energetic	CPET parameters	Peak VO2 < 14–16 mL/kg/min; VE/VCO2 slope > 34	Exercise training, NO-pathway and mitochondrial-targeted therapies

* Thresholds are proposed as pragmatic, biologically informed indicators intended for hypothesis generation and clinical stratification rather than validated diagnostic cutoffs. Abbreviations: AF, atrial fibrillation; BMI, body mass index; CPET, cardiopulmonary exercise testing; EAT, epicardial adipose tissue; eGFR, estimated glomerular filtration rate; HbA1c, glycated hemoglobin; HOMA-IR, homeostatic model assessment of insulin resistance; hs-CRP, high-sensitivity C-reactive protein; MRA, mineralocorticoid receptor antagonist; NO, nitric oxide; NT-proBNP, N-terminal pro-B-type natriuretic peptide; RAAS, renin–angiotensin–aldosterone system; sST2, soluble suppression of tumorigenicity-2; SGLT2, sodium–glucose cotransporter 2; SR, sinus rhythm; VE/VCO2, ventilatory efficiency/carbon dioxide production slope; VO2, oxygen consumption.

## Data Availability

No new data were created.
